# Effectiveness of Simulation-Based Training in Urology: A Systematic Review of Educational Outcomes and Clinical Skill Transfer

**DOI:** 10.7759/cureus.98641

**Published:** 2025-12-07

**Authors:** Maanya Bhardwaj, Abhinav Singhal, Gaurika Bhardwaj, Hariom Sur

**Affiliations:** 1 Urology, Cambridge University Hospitals NHS Foundation Trust, Cambridge, GBR; 2 Urology, University Hospitals Birmingham NHS Foundation Trust, Birmingham, GBR; 3 Urology, Chelsea Westminster Hospital NHS Foundation Trust, London, GBR

**Keywords:** augmented reality, educational outcomes, simulation, simulation-based training, simulation training, skills training, urology resident, urology trainee, virtual reality, vr

## Abstract

Urology is a technically demanding specialty, where training opportunities can be limited by patient safety, working hours, and case availability. Simulation-based medical education (SBME) is being increasingly integrated into urology training, offering opportunities for deliberate practice in a structured, reproducible, and risk-free environment. The effectiveness of modern simulation modalities and the extent of clinical skill transfer, however, remain variably reported. This review aims to evaluate the educational outcomes, validity evidence, and clinical skill transfer associated with contemporary simulation-based training in urology.

A systematic review was conducted in accordance with PRISMA guidelines. PubMed/MEDLINE, EMBASE, Cochrane CENTRAL, and SCOPUS were searched for studies published between 1st January 2015 and 10th November 2025. Eligible designs included randomized trials, cohort studies, and prospective or retrospective evaluations involving urology trainees or consultants. Outcomes of interest were educational performance, technical skill acquisition, and transfer of skills to clinical practice. Risk of bias was assessed using ROBINS-I and the Cochrane Risk of Bias tool. Owing to the heterogeneity of interventions and outcomes, a narrative synthesis was undertaken.

Fifteen studies involving 475 participants met the inclusion criteria. Simulation modalities included virtual reality (VR) platforms, multimodal endourological curricula, laparoscopic simulators, and patient-specific 3D-printed or hydrogel models. High-fidelity simulators demonstrated strong face, content, and construct validity, consistently distinguishing between novice and experienced surgeons. Short-format and proficiency-based progression curricula yielded significant improvements in technical skills. Several studies demonstrated predictive validity, showing that VR-trained participants achieved superior operative performance in procedures such as transurethral resection of bladder tumor, ureteroscopy, and laparoscopic nephrectomy. Patient-specific rehearsal models enhanced anatomical understanding and influenced operative planning for complex cases. Common limitations included small sample sizes, single-center designs, and limited reporting of long-term patient outcomes.

Modern SBME is an effective and validated tool for developing technical proficiency in urology and demonstrates meaningful transferability to clinical practice. Further multicenter studies using standardized outcomes are required to assess long-term patient impact and evaluate the cost-effectiveness of advanced simulation technologies.

## Introduction and background

Simulation is defined as the use of a person, device, or set of conditions that realistically replicate clinical situations for education, training, or assessment, requiring the learner to respond as they would in real practice [1]. Simulation-based medical education (SBME) provides a controlled, risk-free environment that enables repetitive practice and objective assessment, a key principle of modern surgical education [2]. Within urology, a technically demanding specialty where procedural proficiency directly influences patient outcomes, SBME has become a critical adjunct to the traditional apprenticeship model of learning.

Traditional urology training is largely experiential, relying on intraoperative exposure and supervised participation in cases. However, constraints such as limited operating time, patient-safety considerations, variability in case volume, and work-hour restrictions have diminished opportunities for hands-on learning [3]. These challenges have driven the adoption of simulation as a complementary modality that allows standardized, reproducible skill development without compromising patient safety.

SBME encompasses a spectrum of modalities, ranging from low-fidelity bench models to high-fidelity virtual reality (VR), augmented reality (AR), mixed-reality systems, and 3D-printed anatomical models [4-6]. These platforms enable trainees to acquire both technical and non-technical competencies, bridging the gap between theoretical instruction and clinical performance. Beyond technical skill acquisition, SBME also supports cognitive, team-based, and decision-making skills essential for safe surgical practice.

From an educational perspective, the theoretical foundations of SBME are well established. Ericsson’s model of deliberate practice emphasizes repeated, feedback-driven task performance to achieve mastery [7], while Fitts and Posner’s three-stage model describes progression from cognitive understanding to autonomous expert performance [8]. Together, these frameworks explain how simulation enhances both psychomotor skill development and the transfer of learning to real-world surgical contexts [7,8].

Despite widespread adoption, uncertainty remains regarding the magnitude of SBME’s effectiveness and its translation to clinical outcomes in urology. Earlier reviews have primarily focused on general surgical education or older, low-fidelity training tools [9,10]. Moreover, recent technological advances, particularly in immersive and interactive simulation, have outpaced the existing evidence syntheses. This review, therefore, seeks to determine, among urology trainees and practicing urologists, whether SBME using contemporary VR, AR, bench, or 3D-printed platforms improves technical performance, educational outcomes, and transfer of skills to clinical practice when compared with traditional training or no simulation.

## Review

Methods

This systematic review was conducted in accordance with the Preferred Reporting Items for Systematic Reviews and Meta-Analyses (PRISMA) guidelines [11]. A comprehensive search was conducted in the following databases: PubMed/MEDLINE, EMBASE, Cochrane Central Register of Controlled Trials (CENTRAL), and SCOPUS. The search included all articles published between 1st January 2015 and 10th November 2025. A structured search strategy was developed using keywords and Medical Subject Headings (MeSH) terms. The search terms included combinations of "urology trainee," "urology resident," "simulation training," "simulation-based training," "skills training," "virtual reality," "augmented reality," "educational outcomes," "learning outcomes," and "clinical skills." Full search strategies utilized for the databases are available in the Appendices. Titles and abstracts of studies were screened independently by three reviewers using the inclusion and exclusion criteria outlined in Table 1. Full texts were retrieved for eligible studies and assessed for final inclusion.

**Table 1:** Exclusion and inclusion criteria for selecting eligible studies

	Inclusion	Exclusion
Population	Urology trainees, urology consultants	Medical students, other physicians and surgeons, specialist nurses, other healthcare professionals
Study design	Randomised controlled trials, prospective or retrospective cohort studies, case control studies	Case reports, case series, systematic reviews and meta-analysis, editorials, conference abstracts without extractable data
Language	Papers in English	Non-English studies
Time frame	Publications between 1st January 2015-10th November 2025	Studies not in this timeframe
Outcomes	Studies reporting on educational outcomes (knowledge, skills, competence), technical skill acquisition and performance, transfer of skills to clinical practice	Studies not reporting on relevant clinical or educational outcomes

A standardized data extraction form was developed, and extracted variables included study citation, country, study design, participant characteristics, sample size, simulator type, task and procedure evaluated, outcome measures, statistical methods, and principal findings. Three reviewers (AS, MB, and GB) independently extracted data from each included study. Any discrepancies were resolved through discussion and, when necessary, adjudicated by a fourth reviewer (HS).

The methodological quality of included studies was assessed using the ROBINS-I tool for non-randomized studies [12] and the Cochrane Risk of Bias tool for randomized controlled trials [13]. Each domain within these tools was rated as low, moderate, or high risk of bias. Differences in reviewer judgments were addressed through discussion and consensus, with final decisions guided by the predefined inclusion and exclusion criteria. In cases where consensus could not initially be reached, a fourth reviewer provided adjudication.

Due to substantial heterogeneity in study design, interventions, comparators, and outcome reporting, a narrative synthesis was conducted instead of a quantitative meta-analysis. The included studies varied widely in type (validation studies, training interventions, and curriculum evaluations), simulation modality (VR, AR, bench models, 3D printing, and telementoring), and outcome domains (face, content, and construct validity; educational performance; and clinical transferability). Outcome reporting was inconsistent, with many studies lacking standardized effect sizes or variance estimates, precluding statistical pooling. Subgroup analyses by modality (robotic, endoscopic, or mixed reality) and sensitivity analyses excluding studies at high risk of bias were initially considered; however, the limited number of comparable studies and incomplete outcome data rendered these analyses infeasible.

Results

**Figure 1:**
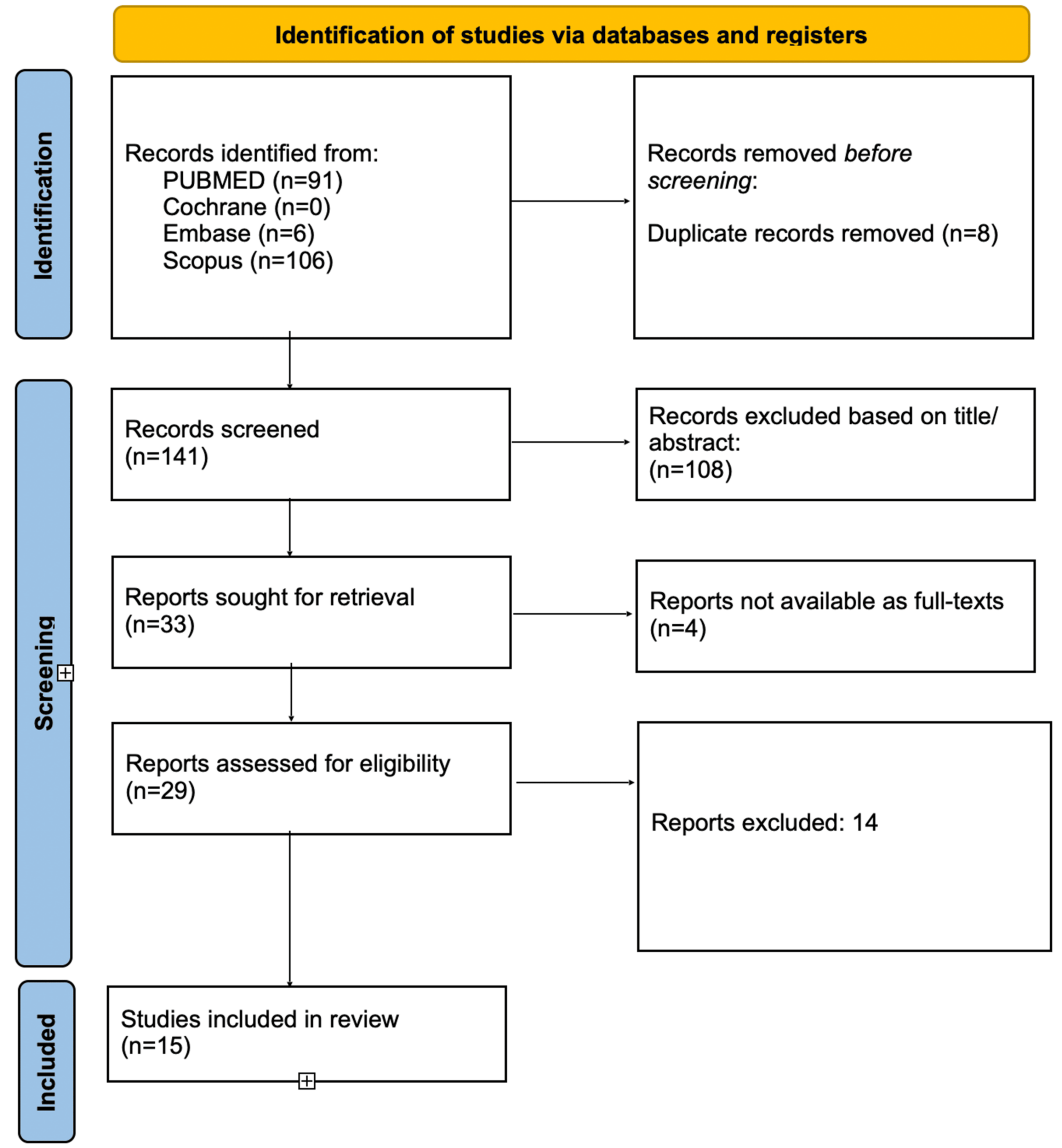
PRISMA flow diagram providing an overview of the selection process for inclusion of eligible studies PRISMA: Preferred Reporting Items for Systematic Reviews and Meta-Analyses

The initial database search yielded 203 records, with 141 remaining after duplicates were removed. Following title and abstract screening, 29 full-text articles were reviewed for eligibility; 15 studies met the inclusion criteria and were included in the final synthesis. Reasons for exclusion at full-text review included unavailable full texts, publications in non-English languages, studies conducted outside the specified timeframe, studies involving medical students or non-urology residents, such as gynecologists or general surgeons, and studies focused on simulator development rather than evaluating their impact on primary outcomes. Figure 1 depicts the PRISMA flow diagram, which summarizes the study selection process.

The eligible studies comprised three randomized controlled trials evaluating VR-based training or planning and 12 non-randomized studies, including validation studies and observational cohort designs. The total number of participants across 15 studies was 475 urologists, primarily consisting of urology residents and fellows, ranging from novices with no prior simulation experience to intermediate trainees. Experienced consultants were frequently enlisted to establish benchmarks for construct validity or to evaluate high-fidelity rehearsal models. The primary procedures investigated were robot-assisted radical prostatectomy (RARP), transurethral resection of bladder tumors (TURBT), and flexible ureteroscopy (URS). Table 2 summarizes the key characteristics of the included studies. The studies employed diverse simulation platforms, including LapSim (Surgical Science, Göteborg, Sweden), UroMentor (Surgical Science, Göteborg, Sweden), RobotiX Mentor (Surgical Science, Göteborg, Sweden), TURBT Mentor (Surgical Science, Göteborg, Sweden), hydrogel-based phantoms, and bespoke 3D-printed simulators. You can also find the comprehensive data extraction from the included studies in the Appendices.

**Table 2:** Summary of key characteristics of included studies PGY: postgraduate year, VR: virtual reality, dVSS: da Vinci skills simulator, FIRST: fundamental interactive robotic surgical tasks, RARP: robot-assistes radical prostatectomy, OR: operating room, TURBEST: transurethral resection of bladder tumors - embedded simulation test, TURBT: transurethral resection of bladder tumor, RCT: randomised controlled trial, PCNL: percutaneous nephrolithotomy, GRS: global rating scale, OSATS: objective structured assessment of technical skills, URS: ureteroscopy, OSATURBS: objective structured assessment for transurethral resection of bladder tumors skills, RAPN: robot-assisted partial nephrectomy

Study	Study design	Participants	Simulator	Procedure	Outcome measures	Main educational outcomes
Carneiro et al., 2022 [14]	Prospective randomized study	36 urology residents and junior staff	Surgical robot skills simulator	Robotic skills	Simulator metrics and task completion	Telementoring improved robotic performance during simulation
Aloosh et al., 2016 [15]	Pilot training and transfer study	PGY 1–4 urology trainees	UroMentor VR	Flexible ureteroscopic stone extraction	VR metrics and operating-room performance checklist	Significant learning curve on VR simulator
Ohtake et al., 2021 [16]	Controlled trial	12 residents (6 training, 6 no training)	LapPASS VR	Laparoscopic nephrectomy	Procedure time, errors, global rating scale	Trained group performed better on simulator
Aghazadeh et al., 2016 [17]	Concurrent validity study	21 surgeons	dVSS + FIRST tasks	Robotic skills; endopelvic dissection in RARP	FIRST/dVSS metrics, intraoperative scores	Demonstrated concurrent validity (simulator–OR correlation)
Bube et al., 2019 [18]	Development and validation	Experts and intermediates	TURBEST	TURBT	Validity testing across experience levels	Construct validity supported
Özkan et al., 2025 [19]	Intensive training evaluation	Participants in hands-on courses	dVSS	Robotic skills (dVSS)	Simulator metrics	Improved simulator performance across sessions
Mittal et al., 2025 [20]	Prospective RCT	90 residents and consultants	Anatomage 3D VR	PCNL planning/training	Accuracy, planning quality, task metrics	VR improved procedural planning
Kim et al., 2015 [21]	Concurrent and predictive validation	11 trainees	Robotic simulator (tube 3 module)	VUA during RARP	Simulator metrics + OR anastomosis quality	Good predictive validity
Soria et al., 2015 [22]	Validation study	60 urologists	Bench + biologic + porcine model	Retrograde intrarenal surgery	Face/content/construct validity	Validated multi-modal RIRS model
Alwaal et al., 2015 [23]	Predictive validity	12 residents	LapSim VR + porcine nephrectomy	Basic laparoscopy; radical nephrectomy	Time, errors, GRS	Construct validity of LapSim
Bendre et al., 2020 [24]	Development + validation	8 residents, 3 faculty	3D-printed silicone pyeloplasty model	Robotic dismembered pyeloplasty	Crowdsourced OSATS scoring	Improved performance after training
Olsen et al., 2021 [25]	Validation study	27 doctors	RobotiX Mentor VR	RARP modules	Simulator metrics (G-theory reliability)	Strong construct validity; reliable metrics
Aydin et al., 2021 [26]	Prospective multi-centre study	46 residents	Multimodal URS simulation curriculum	Ureterorenoscopy	OSATS during training and OR	Improved skills across all modules
Bube et al., 2022 [27]	National implementation study	31 trainees	TURBT mentor VR	TURBT	OSATURBS clinical rating	Novices showed significant improvement
Ghazi et al., 2024 [28]	Pilot study	25 RAPN patients and operating surgeons	Hydrogel 3D-printed kidney phantom	RAPN rehearsal	Planning metrics, surgical decision-making	Improved anatomical understanding and planning

Educational and Technical Performance Outcomes

Across the included studies, VR training consistently improved simulator-derived technical performance. For URS, Aloosh et al. [15] demonstrated significant improvements in VR metrics, demonstrating a clear learning curve. Ohtake et al. [16] reported superior simulated laparoscopic nephrectomy performance in the trained group compared with controls. In robotic simulation training, Carneiro et al. [14] and Özkan et al. [19] documented improvements in time, precision, and overall simulator task completion. Similar gains were observed with TURBT, with Bube et al. [27] reporting substantial improvements among trainees completing a standardized VR curriculum.

Several intensive or structured training formats demonstrated rapid skill acquisition. Özkan et al. [19] reported a 17% improvement across robotic simulation metrics following a three-day course, while Bendre et al. [24] found that trainees achieved higher objective structured assessment of technical skills (OSATS) scores after repeated practice on a 3D-printed pyeloplasty model.

Validity Evidence

Twelve studies reported validity evidence across multiple domains. Construct validity was demonstrated in ten studies, including those assessing TURBT [18,27], URS [26], and robotic surgery tasks [17,21,25]. Six studies supported concurrent validity; for example, Aghazadeh et al. [17] found positive correlations between robotic simulator metrics and intraoperative global evaluative assessment of robotic skills (GEARS) scores, while Olsen et al. [25] demonstrated strong agreement between simulator performance and expert ratings for RARP modules. Predictive validity was more variable. Kim et al. [21] demonstrated that simulator performance predicted vesicourethral anastomosis quality during RARP, whereas Alwaal et al. [23] found no significant correlation between LapSim performance and outcomes in porcine nephrectomy. Studies using automated simulator metrics, such as Olsen et al. [25] and Bube et al. [18], generally showed lower measurement bias than those relying on subjective expert assessments.

Transfer of Skills to Clinical Practice

Nine studies evaluated the transfer of simulation-acquired skills to operative performance, including one randomized controlled trial. In TURBT, Bube et al. [27] reported improved intraoperative objective structured assessment for transurethral resection of bladder tumors skills (OSATURBS) scores following national implementation of a VR curriculum. In URS, Aloosh et al. [15] demonstrated improved operative performance in a pilot transfer study, and Aydın et al. [26] observed significant skill improvements during clinical URS procedures following multimodal simulation training. Evidence for robotic surgery was mixed: Aghazadeh et al. [17] found that simulator metrics correlated with intraoperative GEARS scores during RARP, whereas Ohtake et al. [16] reported that improvements on VR simulators did not consistently predict performance during porcine nephrectomy.

Patient-Level Outcomes

Only two studies assessed patient-level outcomes. Mittal et al. [20] found that VR-enhanced planning for percutaneous nephrolithotomy (PCNL) improved surgical planning accuracy, although effects on operative metrics were modest. Ghazi et al. [28] reported that patient-specific hydrogel kidney phantoms improved preoperative understanding and influenced surgical decision-making during complex partial nephrectomies. However, both studies were small, non-randomized, and susceptible to confounding, resulting in very low certainty regarding patient-level benefit.

Risk of Bias of Included Studies

Overall, the methodological quality of the included studies was judged to be moderate, with notable differences between randomized and non-randomized designs. The three randomized controlled trials [14,16,20] exhibited several concerns regarding unclear random sequence generation and allocation concealment, which limited confidence in group comparability. None of the trials reported blinding of outcome assessors, and two relied partly on subjective rating scales, introducing a high risk of measurement bias. However, the use of objective simulator metrics in some studies reduced outcome-assessor bias, incomplete reporting of trial procedures, and selective outcome reporting, thereby limiting overall methodological robustness.

The 12 non-randomized studies [15,17-19,21-28] were generally at moderate to high risk of bias according to ROBINS-I. The most frequent limitation was confounding, predominantly due to unadjusted differences in baseline surgical experience among participants. Several studies relied on convenience or volunteer sampling, increasing the risk of selection bias. Measurement bias was also typical: in many studies, outcomes were evaluated using subjective expert assessments without blinding, particularly for intraoperative or OSATS-based ratings [15,17,21,26]. Although studies using automated simulator metrics (e.g., Olsen et al. [25], Bube et al. [18]) showed lower measurement bias, these were in the minority. Attrition and incomplete follow-up were noted in several longitudinal or multi-stage training studies, further contributing to potential bias.

Overall, these methodological concerns, especially the lack of blinding and reliance on subjective outcome measures, limit the certainty of the evidence and directly informed the downgrading of outcomes in the grading of recommendations assessment, development, and evaluation (GRADE) assessment. Detailed domain-level ratings for randomized studies and non-randomized studies are presented in Tables 3-4.

**Table 3:** RoB assessment for RCTs using the Cochrane RoB assessment tool GOALS: global operative assessment of laparoscopic skills, VR: virtual reality, CT: computed tomography, PCNL: percutaneous nephrolithotomy, RCTs: randomized controlled trials, RoB: risk of bias

Study	Randomisation process	Deviations from intended interventions	Missing outcome data	Measurement of outcomes	Selection of reported results	Overall RoB	Key notes
Carneiro et al., 2022 [14] (remote vs in-person proctoring)	Moderate	Low	Low	Low	Low	Moderate	Randomisation not clearly described; objective simulator outcomes reduce measurement bias.
Ohtake et al., 2022 [16] (LapPASS VR → porcine nephrectomy)	Moderate	Low	Low	Moderate	Low	Moderate	Assignment not well-detailed; assessor blinding uncertain for GOALS scores.
Mittal et al., 2025 [20] (CT vs CT + VR PCNL planning)	Low	Low	Low	High	Low	Moderate	Allocation concealment done; but outcomes include subjective, unblinded Likert scores → high measurement bias.

**Table 4:** RoB assessment for non-randomized studies using the ROBINS-I tool URS: ureteroscopy, VR: virtual reality, OR: operating room, GEARS: global evaluative assessment of robotic skills, RARP: robot-assisted radical prostatectomy, TURBEST: transurethral resection of bladder tumors simulator-based test, VUA: vesicourethral anastomosis, TURBT: transurethral resection of bladder tumor, OSATS: objective structured assessment of technical skills, RAPN: robot-assisted partial nephrectomy, 3D: three-dimensional, RIRS: retrograde intra-renal surgery, RoB: risk of bias

Study	Confounding	Selection of participants	Classification of interventions	Deviations from intended interventions	Missing data	Measurement of outcomes	Selection of reported result	Overall RoB	Key notes
Aloosh et al., 2016 [15] (URS VR → OR transfer)	High	Moderate	Low	Low	Low	High	Moderate	High–moderate	Experience not adjusted; unblinded single assessor for OR and VR scores → high measurement bias.
Aghazadeh et al., 2016 [17] (Robotic simulation → GEARS in RARP)	High	Moderate	Low	Low	Low	Moderate	Low	High	Experience strongly confounds simulator–GEARS correlations; blinding unclear.
Bube et al., 2019 [18] (TURBEST simulator validation)	Moderate	Moderate	Low	Low	Low	Low	Low	Moderate	Automated metrics reduce measurement bias; some selection and experience confounding.
Özkan et al., 2025 [19] (3-day robotic simulation course)	High	Moderate	Low	Low	Moderate	Low	Moderate	High	Uncontrolled pre–post design; incomplete follow-up.
Kim et al., 2015 [21] (Tube 3 robotic VUA module)	Moderate	Moderate	Low	Low	Low	Moderate	Moderate	Moderate	Non-random allocation; subjective assessments with unclear blinding.
Bube et al., 2022 [27] (National TURBT mastery learning → OR TURBT)	Moderate	Moderate	Low	Low	Low	Low	Low	Moderate	Pre–post design; but video ratings blinded → reduced measurement bias.
Olsen et al., 2021 [25] (RobotiX Mentor RARP test)	Moderate	Moderate	Low	Low	Low	Low	Low	Moderate	Self-reported experience; automated metrics minimize outcome bias.
Aydın et al., 2021 [26] (SIMULATE URS curriculum)	Moderate	Moderate	Low	Low	Moderate	Moderate	Low	Moderate	30% missing OR follow-up; OSATS uses subjective scoring despite partial blinding.
Ghazi et al., 2024 [28] (Hydrogel RAPN rehearsals)	High	Moderate	Low	Low	Low	Moderate	Low	High	Single-arm expert cohort; subjective surgeon ratings unblinded; major confounding.
Bendre et al., 2020 [24] (3D-printed pyeloplasty + GEARS)	Moderate	Moderate	Low	Low	Low	Moderate	Low	Moderate	Crowd GEARS partially blinded; self-confidence scores subjective.
Soria et al., 2015 [22] (RIRS multimodality training)	Moderate	Moderate	Low	Low	Low	Moderate	Low	Moderate	Single-blinded assessor; participants self-selected; subjective scoring.
Alwaal et al., 2015 [23] (LapSim predictive validity)	Moderate	Low–moderate	Low	Low	Low	Moderate	Low	Moderate	Two blinded raters; predictive validity not shown; some experience confounding.

Discussion

This review demonstrates that SBME in urology is supported by strong validity evidence and is effective in enhancing technical skills across robotic, endourological, and laparoscopic procedures. High-fidelity VR simulators and advanced physical models have consistently demonstrated face, content, and construct validity, with several studies applying contemporary frameworks, such as Messick’s model, to confirm that simulation performance reliably distinguishes between novice and experienced surgeons. Intensive, short-format training curricula were shown to produce significant and rapid gains in technical competence, supporting the integration of proficiency-based mastery learning into structured training pathways. Evidence of predictive validity was also observed, with multiple studies demonstrating meaningful transfer of simulation-acquired skills to the operating room, particularly among novice surgeons. Additionally, patient-specific rehearsal and planning tools, such as 3D-printed hydrogel phantoms and VR reconstructions, were shown to enhance surgical preparedness for complex cases and, in some instances, influence intraoperative decision-making.

GRADE Assessment

The certainty of evidence across the included studies was evaluated using the GRADE framework (Table 5). Evidence that VR simulation improves technical performance on simulator-based tasks was judged to be of low certainty, downgraded for study limitations, non-randomized designs [15,17-19,21-28], subjective outcome assessment in several studies [15,17,21,26], and imprecision associated with small sample sizes across the dataset [15,17,21,24]. Evidence supporting simulator validity (construct, concurrent, and predictive validity) was also rated as low certainty, with downgrading primarily due to methodological concerns, including lack of assessor blinding [15,17,21], variations in validity evaluation methods across studies [18,21,25], and inconsistent effect estimates in predictive validity research [17,21,23]. Evidence relating to the transfer of VR-acquired skills to real operative performance was assessed as very low certainty, reflecting serious risks of bias, inconsistency across studies [15,17,26,27], and substantial imprecision arising from small cohorts and heterogeneous operative outcomes. Finally, evidence evaluating patient-level outcomes was rated as very low certainty because only two small, uncontrolled studies contributed data [20,28], both of which were highly susceptible to confounding and imprecision. Overall, while the direction of effect generally favored VR training, confidence in the magnitude and reliability of these effects remains limited.

**Table 5:** Summary of findings and certainty of evidence (GRADE) for key outcomes RCT: randomized controlled trial, TURBT: transurethral resection of bladder tumor, VR: virtual reality, URS: ureteroscopy, GRADE: grading of recommendations assessment, development, and evaluation

Outcome	No. of studies (design)	Findings	Reasons for downgrading	Overall certainty of evidence (GRADE)
Improvement in simulator-based technical performance	15 studies (3 RCTs; 12 observational)	Most studies demonstrated improved task time, movement efficiency, and error reduction following VR training. Effects were consistent across TURBT, URS, and robotic tasks.	Risk of bias: predominantly non-randomised designs with subjective scoring. Imprecision: small sample sizes. Indirectness: minimal. Publication bias: likely in educational research.	Low
Validity of simulation platforms (construct, concurrent, predictive validity)	12 observational studies	Strong evidence for construct validity across modalities; moderate evidence for concurrent and predictive validity, with mixed results in robotic and laparoscopic tasks.	Risk of bias: Limited blinding; unadjusted confounding. Inconsistency: Variability in predictive validity. Imprecision: Small exploratory samples.	Low
Transfer of VR-acquired skills to operative performance	1 RCT + 8 observational studies	Some studies showed improved intraoperative performance (notably TURBT and URS), but findings were inconsistent across procedures and simulation platforms.	Risk of bias: serious confounding; unblinded assessments. Inconsistency: mixed operative outcomes. Imprecision: small, heterogeneous cohorts.	Very low
Impact on patient-level outcomes	2 small observational studies	Limited evidence of improved surgical planning and potential operative benefits, but no robust patient-centred outcome data.	Risk of bias: uncontrolled designs; major confounding. Indirectness: link between simulation and patient outcomes not directly measurable. Imprecision: extremely small samples.	Very low

Comparison With Existing Literature

The results of this review align with broader findings in the surgical education literature, which increasingly support the role of SBME as a complement to traditional apprenticeship-style training. Earlier studies evaluating older laparoscopic simulators reported inconsistent predictive validity; however, the current evidence indicates that modern VR platforms offer improved realism, more accurate metrics, and stronger correlations with intraoperative performance. The observed benefits of patient-specific rehearsal reflect trends in other surgical specialties, where personalized simulation is being adopted to optimize preoperative planning, particularly for complex or high-risk cases. The finding that simulation yields the most significant benefit early in training is consistent with established learning curve theory. It suggests that the value of SBME may diminish as procedural experience accumulates.

Strengths and Limitations of the Evidence

Although the included studies collectively support the effectiveness of SBME, several limitations should be considered when interpreting the results. Many investigations used small sample sizes, single-center cohorts, or non-randomized designs, which may limit generalisability. Considerable heterogeneity existed in simulator platforms, outcome measures, and assessment tools, reducing the comparability of findings across studies. While short-term skill acquisition and transfer to clinical performance were frequently documented, evidence regarding the long-term impact of SBME on patient outcomes, such as complication rates, oncological outcomes, or readmission rates, remains limited. Furthermore, the substantial cost associated with advanced VR technology and patient-specific modelling may restrict accessibility in resource-limited settings.

Limitations of the Review

Several methodological factors may constrain this review. Heterogeneity in study design and outcome reporting across the included randomized and non-randomized studies [14-28] limited the feasibility of conducting a meta-analysis and restricted direct comparisons across simulation modalities. In addition, including only published English-language studies may introduce selection or publication bias, potentially favoring studies reporting positive educational effects. Variability in how validity frameworks were applied across studies, particularly in assessing construct, concurrent, and predictive validity [18,21,25], may also influence the interpretation of evidence strength.

Implications for Practice and Future Research

The findings support the incorporation of SBME into urology training programs, particularly for early learners and for procedures with steep learning curves. Proficiency-based mastery learning and structured VR curricula may help ensure that trainees achieve a baseline level of competence before performing procedures on patients. The evidence further suggests that patient-specific rehearsal may have a valuable role in preparing for complex cases, although widespread adoption will depend on cost-effectiveness and resource availability. Future research should prioritize multicenter, adequately powered studies using standardized outcome measures, as well as investigations assessing the long-term impact of simulation training on patient-centered outcomes. Economic evaluations are also needed to guide the rational implementation of advanced simulation technologies.

## Conclusions

Simulation-based training has matured into an essential component of urological education, offering a validated means to acquire technical skills outside the operating room. The evidence supports the efficacy of proficiency-based curricula for novices, demonstrating clear transferability of skills to clinical practice for procedures including TURBT, URS, and robotic surgery. Furthermore, the advent of patient-specific rehearsal platforms offers significant value for experienced surgeons planning complex interventions. Future research should prioritize multi-institutional trials and focus on determining the impact of simulation training on long-term patient safety and clinical outcomes.

## Appendices

Appendix A: search strategy for various databases

**Table 6:** Search strategy for the databases

Data base	Search strategy	No. of articles
PubMed	(("Urology"[Mesh] OR urologic* OR "urology trainee*" OR "urology resident*") AND ("Simulation Training"[Mesh] OR "simulation-based training" OR "simulation training" OR "skills training" OR simulation) AND ("Virtual Reality"[Mesh] OR "virtual reality" OR VR OR "Augmented Reality"[Mesh] OR "augmented reality" OR AR OR "mixed reality" OR "3D printing" OR "bench model" OR "telementoring") AND ("Educational Measurement"[Mesh] OR "educational outcomes" OR "learning outcomes" OR "clinical skill*" OR "skills transfer" OR "performance" OR "competency"))	91
COCHRANE Library	(urology OR urologic OR "urology trainee" OR "urology resident") AND ("simulation-based training" OR "simulation training" OR simulation OR "skills training") AND ("virtual reality" OR "augmented reality" OR "mixed reality" OR "3D printing" OR "bench model" OR "telementoring") AND ("educational outcomes" OR "learning outcomes" OR "clinical skills" OR "skill transfer" OR "performance" OR "competency" OR "clinical competence") Filters: English language, human studies, 2015–2025.	0
EMBASE	('urology'/exp OR urologic*:ti,ab OR 'urology resident':ti,ab OR 'urology trainee':ti,ab) AND ('simulation training'/exp OR 'skills training':ti,ab OR simulation-based training:ti,ab OR simulation:ti,ab) AND ('virtual reality'/exp OR 'augmented reality'/exp OR 'mixed reality':ti,ab OR VR:ti,ab OR AR:ti,ab OR '3D printing':ti,ab OR 'bench model':ti,ab OR 'telementoring':ti,ab) AND ('educational outcome'/exp OR 'learning outcome'/exp OR 'clinical skill':ti,ab OR 'skills transfer':ti,ab OR 'performance':ti,ab OR 'competency':ti,ab) AND [humans]/lim AND [english]/lim AND (2015-2025)/py	6
SCOPUS	TITLE-ABS-KEY( (urology OR urologic* OR "urology resident*" OR "urology trainee*") AND ("simulation-based training" OR "simulation training" OR simulation OR "skills training") AND ("virtual reality" OR "augmented reality" OR "mixed reality" OR "3D printing" OR "bench model" OR telementoring) AND ("educational outcomes" OR "learning outcomes" OR "clinical skills" OR "skill transfer" OR performance OR competency OR "clinical competence") ) AND (LIMIT-TO (LANGUAGE, English)) AND (PUBYEAR > 2014 AND PUBYEAR < 2026)	106

Appendix B: comprehensive data extraction from included studies

**Table 7:** Comprehensive data extraction from included studies PGY: postgraduate year, RW2: ring walk 2, ED2: energy dissection 2, RW3: ring walk 3, VR: virtual reality, dVSS: da Vinci skills simulator, FIRST: fundamental interactive robotic surgical tasks, RARP: robot-assisted radical prostatectomy, OR: operating room, TURBEST: transurethral resection of bladder tumours - embedded simulation test, TURBT: transurethral resection of bladder tumor, PCNL: percutaneous nephrolithotomy, GRS: global rating scale, OSATS: objective structured assessment of technical skills, URS: ureteroscopy, OSATURBS: objective structured assessment for transurethral resection of bladder tumours skills, RAPN: robot-assisted partial nephrectomy, GOALS: global operative assessment of laparoscopic skills, GEARS: global evaluative assessment of robotic skills, 3D: three-dimensional, CT: computed tomography, VUA: vescicourethral anastomosis, RIRS: retrograde intra-renal surgery

Study	Participants (number analysed/level)	Design and comparator	Simulator and procedure	Simulation exposure	Follow-up setting	Primary outcomes and key numerical results
Carneiro et al., 2022 [14]	36 urology residents/junior staff	Prospective 3-arm trial (no proctor vs in-person vs remote proctor)	dVSS; robotic basic skills	3 repetitions each of RW2, ED2, RW3 in one session	Simulator only	ED2 improved 52.8→80.6% (p=0.002); RW2 63.9→83.3% (p=0.032); RW3 differed across groups (p=0.042). Remote/in-person proctoring significantly better than no-proctor.
Aloosh et al., 2016 [15]	13 residents (PGY1–4): 8 in VR training phase, 5 followed in OR	Prospective cohort with OR transfer assessment	UroMentor VR; flexible URS stone extraction	3 weekly sessions; mean 6.4 ± 3.1 repetitions (total ≈52 VR runs)	Operating room	VR competency plateaus after 7 trials. Strong predictive validity: simulator–OR URS-GRS correlation r=0.90 (p=0.03).
Ohtake et al., 2022 [16]	12 residents (6 VR training, 6 control)	Non-randomised controlled trial	LapPASS VR + live porcine laparoscopic nephrectomy	4–5 hours VR training across all basic tasks	Live porcine nephrectomy (single case per trainee)	GOALS efficiency score higher in VR group (p=0.030). Performance variability lower (p=0.005).
Aghazadeh et al., 2016 [17]	21 surgeons (trainees + consultants)	Concurrent-validity cross-sectional study	dVSS and FIRST robotic tasks; real RARP	Single simulator assessment	Operating room (RARP)	Strong correlations: dVSS–GEARS ρ=0.805 (p<0.001); FIRST–GEARS ρ=0.833 (p<0.001).
Bube et al., 2019 [18]	49 urologists grouped by experience	Construct-validation cross-sectional study	TURBEST VR TURBT test	Single test attempt	Simulator only	Mean scores: novice 15.9, intermediate 25.6, expert 30.6; ANOVA p<0.001. Cronbach α≈0.85.
Özkan et al., 2025 [19]	52 robotic trainees	Pre–post cohort (no comparator)	Robotic VR simulator (mimic/dV-Trainer)	3-day intensive course; repeated sets of 4 core exercises	Simulator only	Significant improvement across all metrics (box-plot data). Majority improved in ≥3 tasks.
Mittal et al., 2025 [20]	90 PCNL patients (CT only n=45 vs CT + VR n=45)	Randomised controlled trial (planning intervention)	Anatomage VR 3D CT reconstruction	Single pre-operative VR planning session	Operating room PCNL cases	Residents’ image-understanding improved, e.g., location 6.05→8.40 (p<0.001). Consultants improved in complex cases (p≤0.028). No significant OR differences in operative time or complications.
Kim et al., 2015 [21]	11 novices (learning curve) + 10 in validation phase	Learning-curve plus validation (trained vs untrained)	dV-trainer Tube 3 robotic VUA module	1h/day × 7 days; ~41 repetitions to proficiency	Dry-lab VUA model (robotic bench setup)	Needle pass time 323 vs 479 s (p=0.016). VUA time 774 vs 1002 s (p=0.009). End-product score 16.0 vs 8.2 (p=0.009).
Bube et al., 2022 [27]	≈30 urology trainees	National before–after implementation	TURBEST VR curriculum	Mastery learning until passing cut-score	Operating room TURBT	OR TURBT quality improved: higher OSATURBS ratings and more complete resections (exact means available in paper).
Olsen et al., 2021 [25]	27 surgeons (novice → expert continuum)	Construct-validation	RobotiX mentor VR RARP test	Single structured test	Simulator only	Significant discrimination across levels (p<0.001). Score correlated with case volume (r≈0.74). Proposed pass/fail cut-scores.
Aydın et al., 2021 [26]	46 residents (70% OR follow-up)	Prospective cohort (SIMULATE URS curriculum)	Multimodal (VR, bench, cadaver) URS curriculum	5 formal training sessions	Operating room URS (semi-rigid and flexible)	OR OSATS improved: semi-rigid URS p=0.0006; flexible URS p=0.0003. High trainee-rated educational value.
Ghazi et al., 2024 [28]	25 patients/4 surgeons	Prospective pilot	Patient-specific 3D hydrogel RAPN models	One full RAPN rehearsal per case	Live RAPN surgery	Confidence scores ↑ significantly (e.g., 49→93, p<0.004). Strong rehearsal→OR correlations: WIT r=0.81 (p<0.002), EBL r=0.69 (p<0.05).
Bendre et al., 2020 [24]	11 (8 residents, 3 faculty)	Feasibility + validation	3D-printed silicone pyeloplasty model	Two robotic pyeloplasty simulations per person	Simulator only	Attendings faster (22.4 vs 58.1 min, p=0.005). GEARS stable; depth perception improved (p=0.006).
Soria et al., 2015 [22]	60 trainees + 15 experts	Construct-validation, multimodal	Bench model + ex vivo porcine + porcine RIRS	16 hours over 2 days	Animal models	GRS 11.8→27.2 (p<0.0001); checklist 3.97→6.93 (p<0.0001). Experts far exceeded trainee baseline.
Alwaal et al., 2015 [23]	12 residents	Predictive-validity study	LapSim VR (laparoscopy)	3 VR sessions	Porcine nephrectomy	Simulator performance did not significantly predict porcine operative performance.
